# The micromechanics of lung alveoli: structure and function of surfactant and tissue components

**DOI:** 10.1007/s00418-018-1747-9

**Published:** 2018-11-02

**Authors:** Lars Knudsen, Matthias Ochs

**Affiliations:** 1 Institute of Functional and Applied Anatomy, Hannover Medical School, Carl-Neuberg-Str. 1, 30625 Hannover, Germany; 2 Biomedical Research in Endstage and Obstructive Lung Disease Hannover (BREATH), Member of the German Center for Lung Research (DZL), Hannover, Germany; 3 REBIRTH Cluster of Excellence, Hannover, Germany

**Keywords:** Acinus, Type II alveolar epithelial cell, Surfactant, Connective tissue, Acute lung injury, Fibrosis

## Abstract

The mammalian lung´s structural design is optimized to serve its main function: gas exchange. It takes place in the alveolar region (parenchyma) where air and blood are brought in close proximity over a large surface. Air reaches the alveolar lumen via a conducting airway tree. Blood flows in a capillary network embedded in inter-alveolar septa. The barrier between air and blood consists of a continuous alveolar epithelium (a mosaic of type I and type II alveolar epithelial cells), a continuous capillary endothelium and the connective tissue layer in-between. By virtue of its respiratory movements, the lung has to withstand mechanical challenges throughout life. Alveoli must be protected from over-distension as well as from collapse by inherent stabilizing factors. The mechanical stability of the parenchyma is ensured by two components: a connective tissue fiber network and the surfactant system. The connective tissue fibers form a continuous tensegrity (tension + integrity) backbone consisting of axial, peripheral and septal fibers. Surfactant (surface active agent) is the secretory product of type II alveolar epithelial cells and covers the alveolar epithelium as a biophysically active thin and continuous film. Here, we briefly review the structural components relevant for gas exchange. Then we describe our current understanding of how these components function under normal conditions and how lung injury results in dysfunction of alveolar micromechanics finally leading to lung fibrosis.

## The structural components for gas exchange

In biology, function follows form and form follows function. The mammalian lung is a paradigmatic example for this principle. It is a complex organ whose functional capacity as a gas exchanger is directly determined by its microstructure (for review, see Weibel 1984, 2017; Ochs and Weibel 2015; Ochs et al. 2016; Hsia et al. 2016). In humans, at the end of a deep inspiration, over 80% of the total volume of the lung is air, and about 10% is blood. Thus, less than 10% (only a few 100 g) is made of “real” tissue. This tissue consists of more than 40 cell types, originating from all three germ layers, and a sophisticated connective tissue network. Together they form an organ with a complex architecture optimized to serve its main function.

Gas exchange takes place in lung alveoli. The alveolar region (parenchyma) of the lung comprises about 90% of its total volume. The remaining non-parenchyma consists of conducting airways (which are part of the anatomic dead space) and larger vessels. The airways branch in irregular dichotomy into the lung together with the arteries, thus defining broncho-arterial units “inside-out” (from the hilum to the periphery). In the most distal branching generations, alveoli are connected to the airways. Clusters of alveoli are arranged in functional units termed acini. An acinus is defined as the blind-ending parenchymal unit beginning with a transitional (i.e. the first generation of an alveolated) bronchiole. Within an acinus, all airways (alveolar ducts and, depending on the species, respiratory bronchioles) have alveoli attached to their walls and thus participate in gas exchange. Actually, the “wall” of alveolar ducts consists of nothing more than a network of alveolar openings. In the human lung there are around 30,000 acini (reviewed in Weibel et al. 2005).

The inter-alveolar septum provides the structural basis for gas exchange in the lung (see Weibel 1973; Weibel and Gil 1977; Maina and West 2006). It separates the air compartment (alveolar airspace) from the blood compartment (capillary lumen). The design of the inter-alveolar septum must meet certain structural requirements for efficient diffusion of oxygen and carbon dioxide between air and blood, in particular providing a large surface area (in the human lung about 140 m^2^) and a thin diffusion barrier (in the human lung about 2 µm) (Gehr et al. 1978). Moreover, it must also meet the functional requirements of stability (thereby preventing over-distension or collapse of alveoli) and flexibility (thereby being able to follow the movements of the thorax during the breathing cycle). Lung alveoli are mechanically stabilized by two factors: the pulmonary surfactant system and the lung´s connective tissue backbone (see below).

**Fig. 1:**
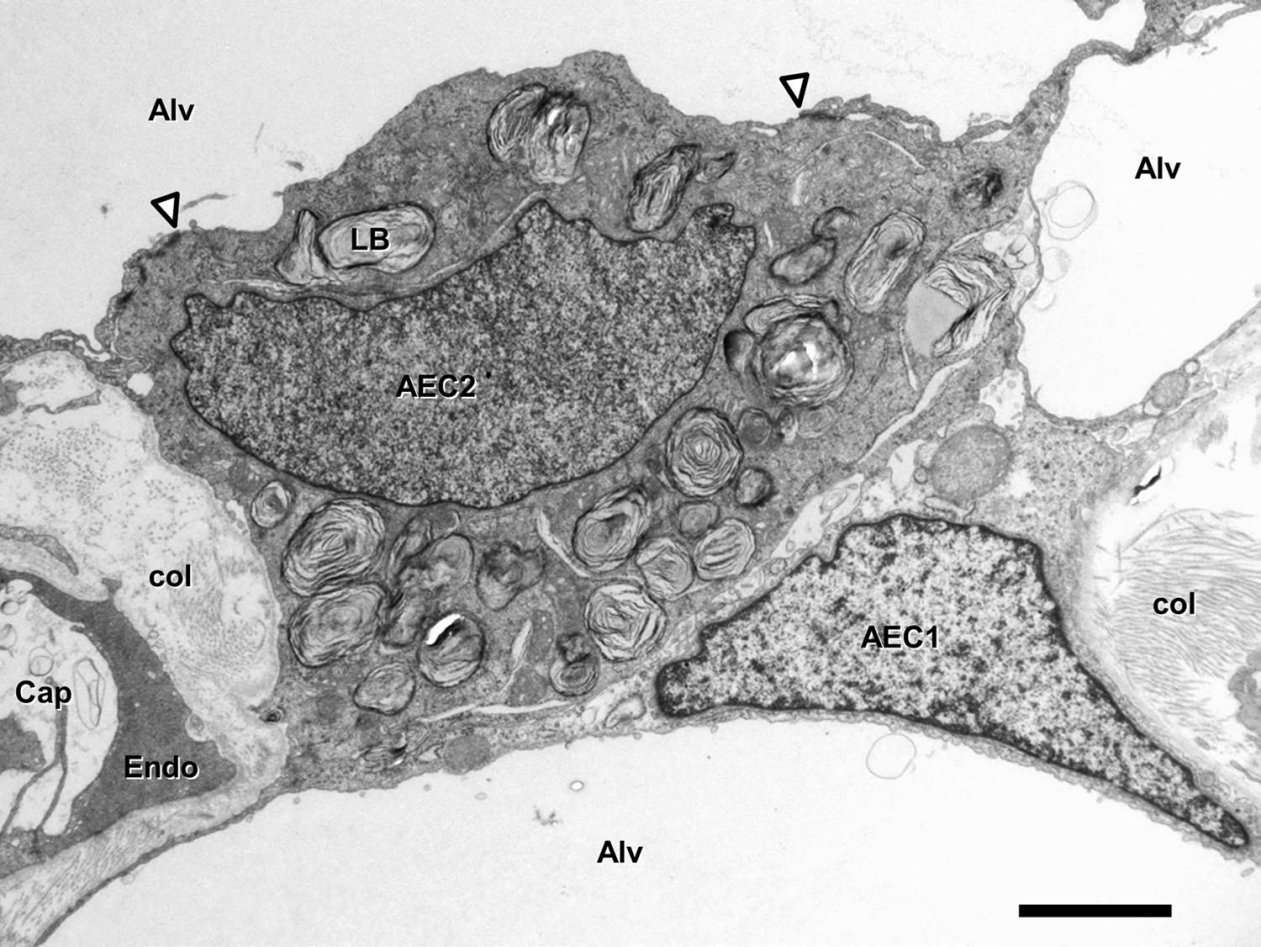
Transmission electron microscopy. Human lung. Inter-alveolar septum with type I (AEC1) and type II (AEC2) alveolar epithelial cell. Note surfactant-storing lamellar bodies (LB) in type II cell. Arrowheads mark tight junctions between type II cell and neighbouring type I cell extensions. Collagen fibrils (col) are present in the interstitium. *Alv* alveolar lumen, *Cap* capillary lumen, *Endo* capillary endothelial cell. Scale bar 2 µm

Within the inter-alveolar septum, the tissue barrier separating air and blood consists of two continuous cell layers: an epithelium facing the alveolar lumen and an endothelium facing the capillary lumen. Between them is an interstitial space of varying thickness and composition. The alveolar epithelium is a mosaic of type I alveolar epithelial cells which cover around 95% of the total alveolar surface with their thin squamous cell extensions interspersed with single cuboidal type II alveolar epithelial cells which are easily recognized by their characteristic secretory organelles, the surfactant-storing lamellar bodies (Fig. 1). Thus, there are two alveolar epithelial cell types with distinct functional specialization and thus distinct structural differentiation. Renewal and repair are provided by the type II cells which, in addition to their secretory function, constitute the progenitor cell population of the alveolar epithelium. The numerical ratio of type I to type II cells is about 1:2 (Crapo et al. 1982). Both type I and type II cells may face more than one alveolar lumen, i.e. they are able to cross the inter-alveolar septum thereby contributing their lining (type I cells) or secretory (type II cells) functions to both sides of the inter-alveolar septum. This level of structural (and thereby functional) complexity has so far not been taken into account in any of the in vitro models of the air-blood barrier. The continuity and tightness of the alveolar epithelium and the capillary endothelium are essential for fluid balance in the alveolar region as these tissue layers separate (and actively regulate) extracellular fluid compartments: blood plasma, interstitial fluid and alveolar lining fluid.

**Fig. 2:**
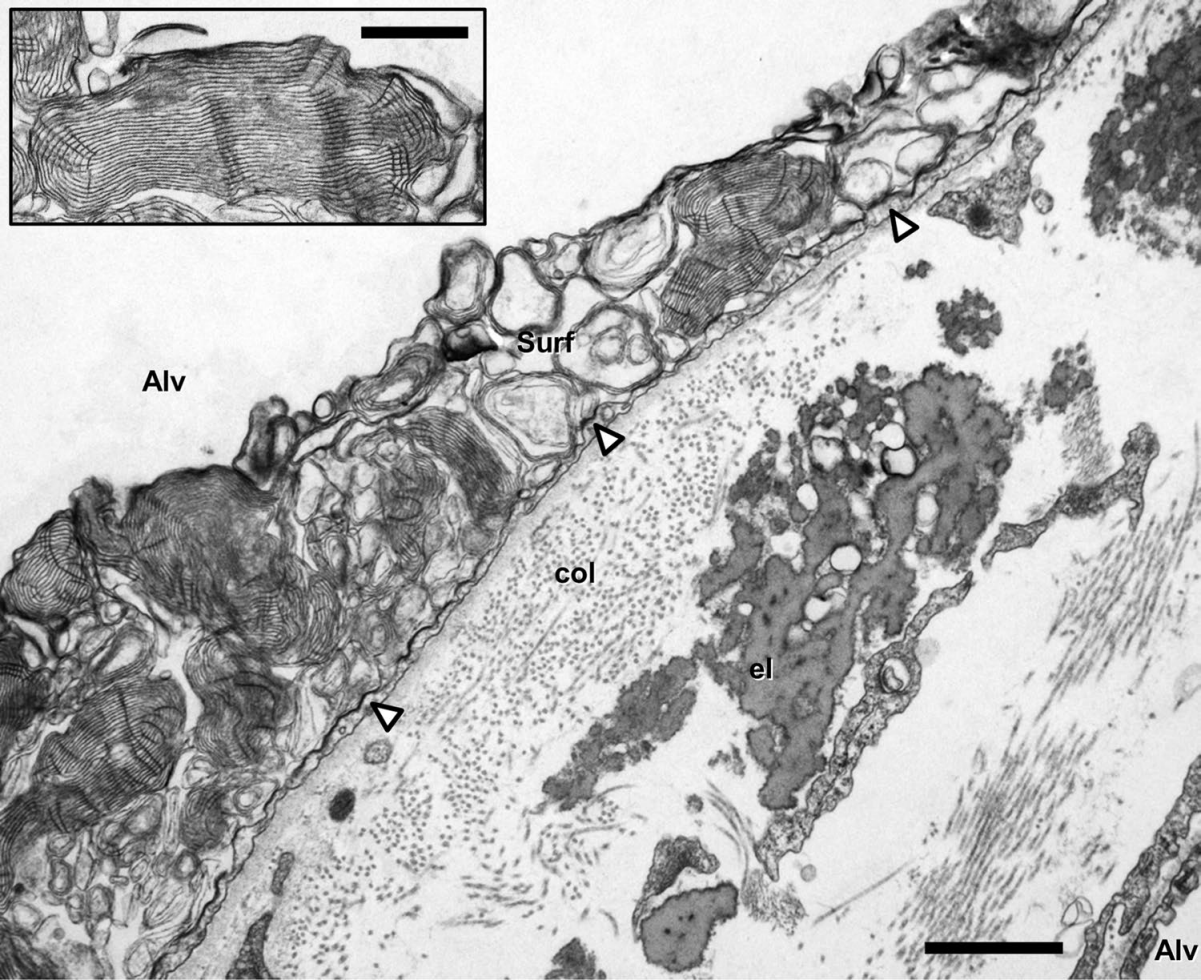
Transmission electron microscopy. Human lung. Inter-alveolar septum with collagen fibrils (col) and elastic fibers (el). The alveolar epithelium (thin type I cell extension marked by arrowheads) is covered with a lining layer containing intra-alveolar surfactant (Surf). *Alv* alveolar lumen. Scale bar 1 µm. Inset shows tubular myelin, a surface-active intra-alveolar surfactant subtype, at higher magnification. Scale bar 0.5 µm

Careful electron microscopic studies revealed the existence of a thin and continuous alveolar lining layer consisting of a surface film and an aqueous hypophase (Weibel and Gil 1968; Gil and Weibel 1969/70). This means that the alveolar epithelium is not directly exposed to air but covered by a liquid lining layer with an estimated mean thickness of about 200 nm in the rat lung (Bastacky et al. 1995). Surfactant is present in the hypophase and constitutes the surface film at the air–liquid interface (for review, see Perez-Gil 2008; Ochs 2010; Ochs and Weibel 2015; Olmeda et al. 2017). All surfactant components (about 90% lipids, mainly saturated phospholipids, and about 10% proteins, including the surfactant proteins SP-A, SP-B, SP-C and SP-D) are synthesized, stored, secreted and to a large extent recycled by type II alveolar epithelial cells (Fig. 1). Most of the intracellular surfactant (at least lipids and the hydrophobic SP-B and SP-C) is assembled in specific organelles, the lamellar bodies, prior to secretion. Intra-alveolar surfactant includes the surface film and different subtypes in the hypophase that can be distinguished morphologically (Fig. 2). Interestingly, these morphologically distinct subtypes largely correspond to different stages in surfactant metabolism and activity. Freshly secreted lamellar bodies transform into tubular myelin, which may act as precursor of the surface film at the air–liquid interface although additional multilayered surface-associated reservoirs have been suggested. “Spent” surfactant is usually present as small unilamellar vesicles which can be taken up by type II cells for recycling or degradation or by alveolar macrophages (the “vacuum cleaners” within the hypophase) for degradation. Overall, surfactant has biophysical as well as immunomodulatory functions. In particular, surfactant stabilizes alveolar dimensions and thus prevents alveolar collapse by a surface-area dependent reduction of alveolar surface tension and is, therefore, essential for normal alveolar micromechanics and lung function (see below).

**Fig. 3:**
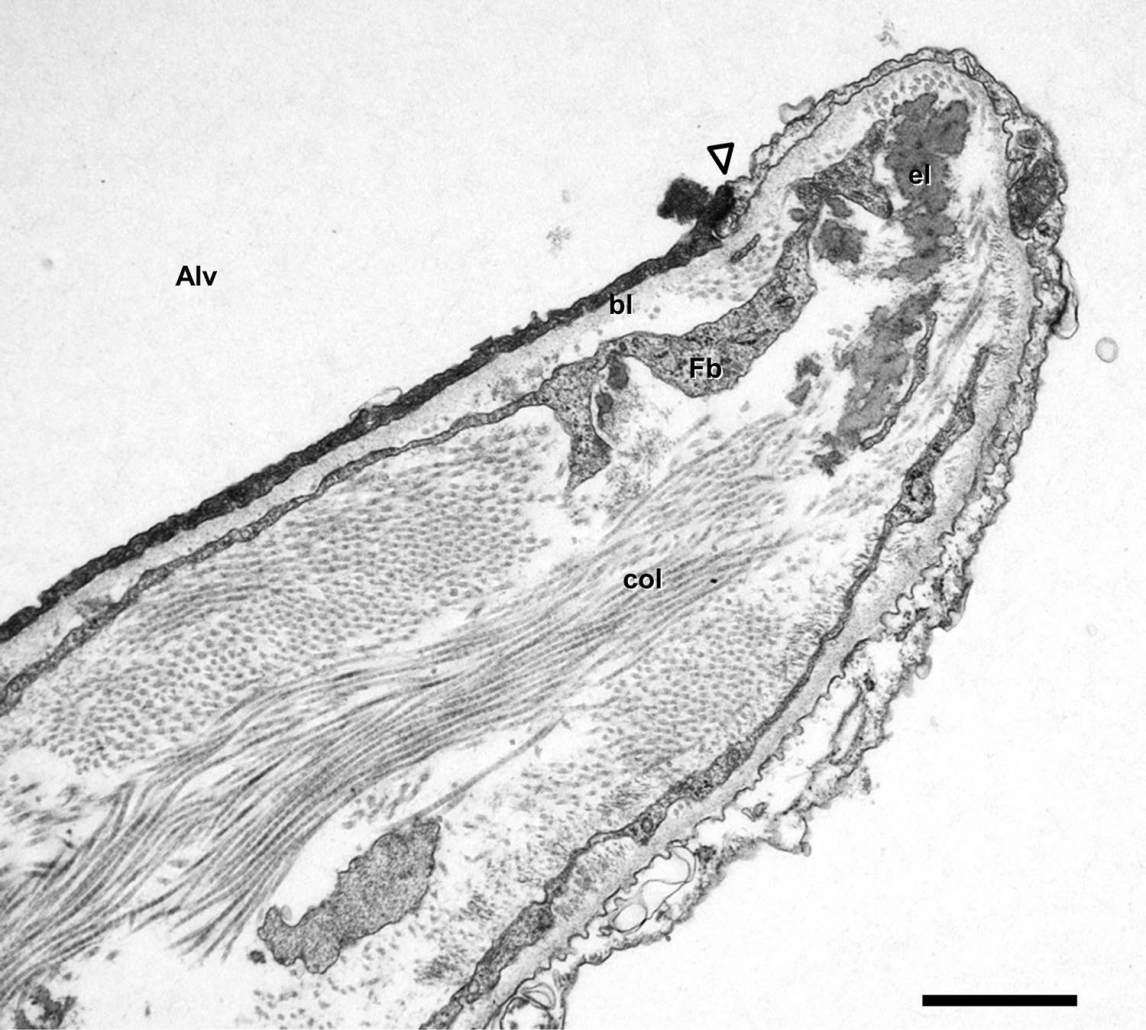
Transmission electron microscopy. Human lung. Inter-alveolar septum with free edge (right) indicating the opening into an alveolar lumen (Alv). Note reinforced entrance ring with elastic fibers (el) at the alveolar opening where the axial fibers are connected to the septal fibers. *col* collagen fibrils, *Fb* fibroblast extensions, *bl* alveolar epithelial basal lamina, arrowhead tight junction between type I alveolar epithelial cells. Scale bar 1 µm

The interstitium, i.e. the bounded space between the alveolar epithelial and capillary endothelial basal laminae, contains cells and an extracellular network of elastic fibers and bundles of banded collagen fibrils forming fibers (Weibel and Crystal 1997). The most abundant cells in the interstitium are the fibroblasts. They are a heterogeneous cell population. While the “classical” fibroblasts produce and maintain the extracellular matrix, many of them have mainly contractile properties. These myofibroblasts contain filaments oriented across the inter-alveolar septum, thus connecting the two epithelial sides of the septum and bracing the interstitial space (Kapanci et al. 1974). Through pores in the basal lamina, myofibroblasts are able to directly link alveolar epithelium and capillary endothelium (Sirianni et al. 2003). The extracellular connective tissue fiber network is interwoven with the alveolar capillary network (Weibel and Crystal 1997; Weibel and Bachofen 1997). Thereby, the air-blood barrier has thick parts where cell nuclei and the fiber network are concentrated (thus providing regenerative capacity and mechanical stability) and thin parts where alveolar epithelium and capillary endothelium share one common basal lamina (thus preventing fluid accumulation and minimizing the thickness of the diffusion barrier to considerably less than 1 µm). In the human lung, about half of the total barrier surface is thin (Weibel 1973; Weibel and Gil 1977). According to its location, the connective tissue network can be subdivided into axial, peripheral and septal fibers (Weibel and Gil 1977; Weibel 2009). Axial fibers enwrap airways from the hilum where the main bronchus enters the lung down to the alveolar ducts where they form a network of opening rings into alveoli. Peripheral fibers extend from the visceral pleura into interlobular septa, thus demarcating broncho-arterial units “outside-in” (from the periphery to the hilum). The septal fibers are anchored to both axial (at the alveolar entrance rings) and peripheral (at the boundary of acini) fibers, thereby constituting a fiber continuum throughout the lung. This continuous network of connective tissue forms a self-stabilizing tensegrity (tension + integrity) structure in the lung (Weibel 2009; Ingber 2003). Of particular interest for alveolar micromechanics are the free edges of the inter-alveolar septa where the axial fiber system of alveolar ducts is connected to the septal fibers of alveoli (Wilson and Bachofen 1982). At these alveolar entrance rings (which also represent the “wall” of alveolar ducts), connective tissue (mostly elastic) fibers and smooth muscle cells can be found (Fig. 3). Air within distal acinar airspaces is either present inside alveolar entrance rings (alveolar airspace) or outside (ductal airspace). Gas exchange is only possible within the alveolar airspace with its capillarized septa. Thus, the ratio of alveolar airspace vs. ductal airspace is highly relevant for proper lung function (see below).

The total surface area of the alveolar capillary endothelium matches that of the epithelium. The individual endothelial cell, however, covers a much smaller surface as compared to its epithelial counterpart, the type I cell (Crapo et al. 1982). This is because endothelial cells have a less complex branching architecture than type I cells which possess several cytoplasmic plates lining the inter-alveolar septum (Weibel 2017). The structure of the alveolar capillary network is considerably different from those of the systemic circulation (Weibel 1963; Mühlfeld et al. 2010; Townsley 2012). Its individual segments form small loops (where the length of the segments is in the range of the capillary diameter), thus resulting in a dense meshwork with vertical tissue pillars in-between. This provides the structural basis for the sheet-flow concept in alveolar capillaries (Fung and Sobin 1969). Because the arteries in the lung follow the conducting airways and their branching in the center of broncho-arterial units whereas the veins run in the connective tissue septa separating them, blood flow is directed from the center to the periphery in these units. In the early postnatal phase a double capillary network layer (one for each alveolar lumen) exists in inter-alveolar septa. After microvascular remodeling, only a single layer is present in the adult lung. As a consequence, this single layer exchanges oxygen and carbon dioxide with two adjacent alveoli. When the septal fibers that are interwoven with this single capillary layer are stretched, the capillaries are spread over both alveolar surfaces of the inter-alveolar septum in a zig-zag pattern, thus maximizing the contact area between air and blood with as little interstitial connective tissue as possible (Weibel and Gil 1977; Ochs and Weibel 2015).

## Function under normal conditions

During the respiratory cycle, the distal airspaces of lung parenchyma are continuously subjected to volume changes. These volume changes impose deformation on ductal and alveolar airspaces and most importantly on inter-alveolar septa. Such deformations can best be described with the term strain which is the size (e.g. length, surface or volume) of a structure after deformation in relation to the baseline situation (Vlahakis and Hubmayr 2005). At the organ scale, the strain imposed on the lung is accordingly calculated using the tidal volume, corresponding to the lung deformation, and the functional residual volume, corresponding to the baseline situation of the lung. During mechanical ventilation the tidal volume is given by the ventilator while the functional residual volume equals the volume of the lung at a given positive end-expiratory pressure (PEEP). Stress, on the other hand, is defined as the force per area so that stress and pressure have the same unit (Vlahakis and Hubmayr 2005). At the microscopic level, pressure-change related alterations of the microarchitecture and, therefore, deformation have been described in ductal and alveolar airspaces as well as inter-alveolar septa (Gil et al. 1979; Bachofen et al. 1987; Tschumperlin and Margulies 1999; Roan and Waters 2011). Based on these observations, it makes physiological sense to distinguish ductal from alveolar airspaces since from an anatomical point of view they are differently bordered with partly different stabilizing elements which result in different mechanical properties (Wilson and Bachofen 1982; Haefeli-Bleuer and Weibel 1988).

### Extracellular matrix, cells and micromechanics of ductal and alveolar airspaces

**Fig. 4:**
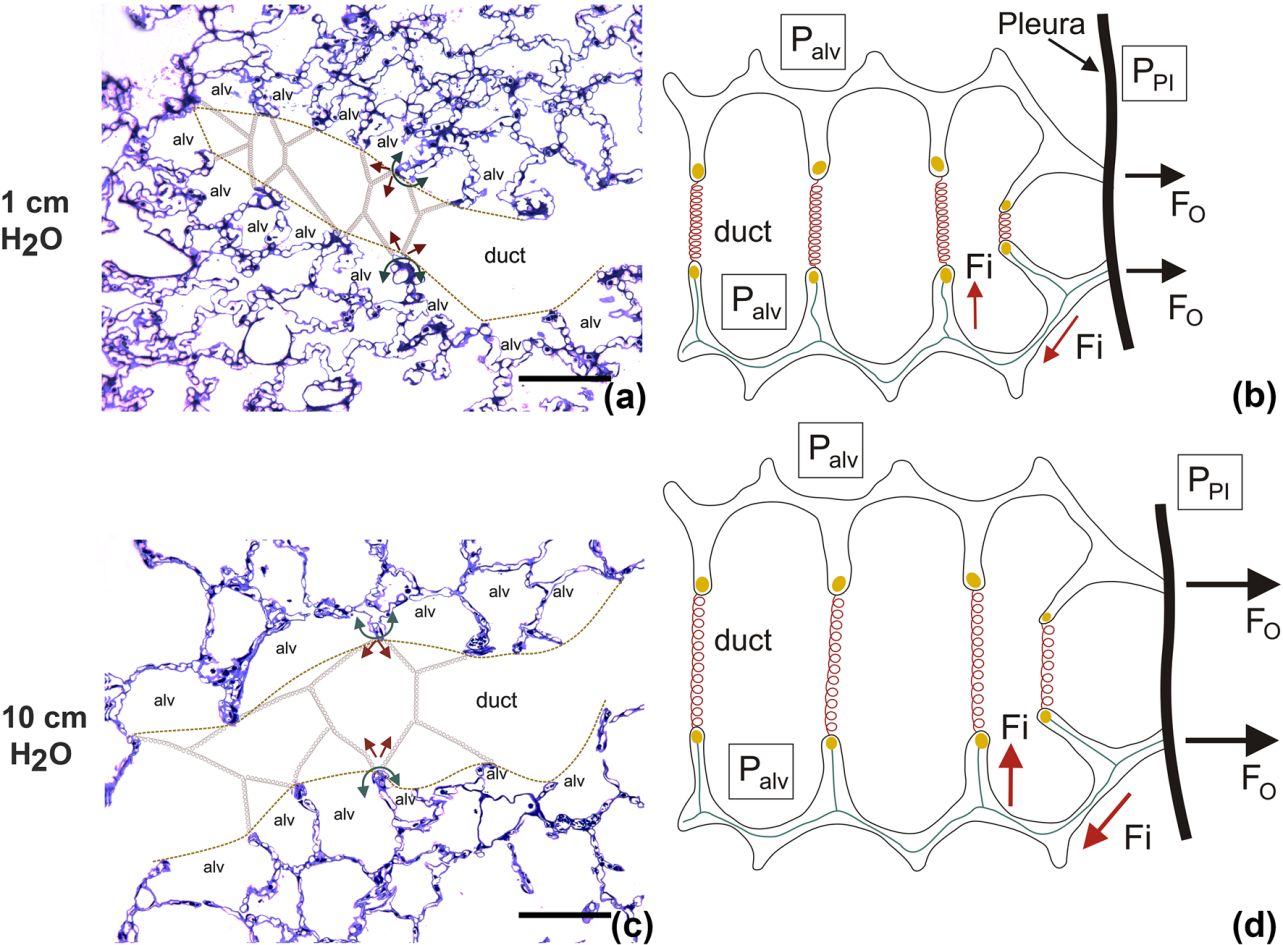
The stress-bearing elements of acinar airspaces. In a previous study (Knudsen et al. 2018), healthy rat lungs were fixed in vivo at airway opening pressure (Pao) of 1 (**a**) and 10 cm H_2_O (**b**). At low pressure, the alveolar ducts are narrow and the inter-alveolar septal walls are characterized by foldings and pleats. The septal walls protrude into the alveolar duct and are connected to the duct via the alveolar entrance. By drawing a straight line between the edges of the septal walls, alveolar and ductal airspaces were separated from each other (fine dashed lines). The axial network of elastic and collagen fibers is concentrated at the edges of alveolar septa and coils the alveolar duct. Here, this system is illustrated as springs spanning the alveolar duct (red springs). At low Pao (or lung volume), the elastic fibers are only slightly stretched (**a, b**). The fibers exert pulling forces on the alveolar edges/entrance rings in the direction of the ductal lumen (red arrows in **a, c**) and counteract the surface tension forces (green arrows in **a, c**) which would pull the septal wall away from the duct and result in a piling up and finally collapse of airspaces. At Pao = 10 cm H_2_O the alveolar duct is widened, the axial fiber system stretched (red springs in c and d). The forces which are responsible for inflation of the lung are related to the pressure gradient between the pleural space (*P*_Pl_) and the alveolar space (*P*_alv_). The outward forces (*F*_O_) are transmitted to the fiber system in the septal walls and correspond here to the inward forces (*F*_i_). Depiction is based on models of Wilson and Bachofen (1982) and Mead et al. (1970). Scale bar 100 µm

An economically designed fiber network serves the stabilization of distal airspaces bearing and transmitting the stresses related to the elastic recoil pressure of the lung (= trans-alveolar pressure). The latter is defined as the difference between the pressure in the acinar airspaces and the pleural surface (Loring et al. 2016). In vivo, the pressure at the pleural surface compared to atmospheric pressure, is usually negative and is mainly based on the elastic recoil of the lung which has its foundation in the elastic fiber network and the surface tension (Fredberg and Kamm 2006; Wilson and Bachofen 1982). The axial system of elastic and collagen fibers originates from the walls of the conducting airways, enters the centers of the acini and contributes to the formation of alveolar entrance rings thereby surrounding the alveolar ducts. Hence, the alveolar duct as such does not have a wall of its own but is instead bounded by the entrance rings of the alveoli which include the elements of the axial network of connective tissue fibers. In other words, the axial fiber system coils the ductal airspaces (Fig. 4). Therefore, volume changes of the alveolar ducts result primarily in a deformation of the alveolar entrance rings and stretch of the axial fiber system. In this context, it has been observed that with decreasing lung volume, the diameter of alveolar entrance rings becomes smaller (Mercer et al. 1987). The alveolar walls, however, are formed by inter-alveolar septa which include above all an alveolar capillary network, diverse cell types and a minimum of stabilizing connective tissue elements. The axial system of connective tissue fibers, concentrated at the alveolar entrance rings, is connected with the peripheral system originating from the pleura by alveolar septal wall fibers which are located between the basal laminae of the alveolar epithelium and endothelium, corresponding to the thick side of the air-blood barrier. These fibers in the thick side represent the backbone of the inter-alveolar septa and transmit the distending forces which in case of homogenous stress distribution within the lung are generated by the pressure gradients between acinar airspace and pleural cavity (Mead et al. 1970) (Fig. 4). With this regard it has been shown that the volume densities of collagen and elastic fibers within the septa increase towards the free edge of the septa forming the “wall” of the alveolar duct (Mercer and Crapo 1990; Toshima et al. 2004). Elastic fibers have a linear stress–strain relationship over a wide range of deformation allowing a doubling of its baseline length (= 200% strain) so that these fibers contribute to elastic recoil and stabilization of lung parenchyma at lower lung volumes including also the range of normal breathing, usually defined as the volume spectrum between 40 and 80% of total lung capacity (TLC) (Suki et al. 2011; Yuan et al. 2000). Collagen fibers, on the other hand, have a more or less curly run at low lung volumes. As a consequence, collagen fibers become straight at larger lung volumes and are then characterized by a highly non-linear stress–strain relationship and high rigidity (Suki et al. 2005).

The stabilizing components of the inter-alveolar septa and, therefore, of the alveolar airspaces also include the alveolar epithelial basal lamina which is supposed to become also stress bearing at larger lung volumes (Maina and West 2006). An increase in volume of the alveoli can result in a stretch of the alveolar epithelial cells which are fixed to the basal lamina via cell-matrix adhesions (Tschumperlin and Margulies 1999). This represents an important difference compared to the alveolar duct which lacks a boundary with a cohesive epithelial lining. However, electron microscopic evaluations of the basal lamina at the thin side of the air-blood barrier of inter-alveolar septa demonstrated folding even at larger lung volumes (e.g. above 80% of TLC) indicating that at least in some areas the basal lamina (and the covering cells) are not entirely stretched (Bachofen et al. 1987). In the healthy lung, these very economically organized stabilizing systems of connective tissue elements described above, allow volumes to change during respiration with minimal effort and without interfering with the parenchyma’s crucial gas exchanging function (Weibel et al. 1991; Weibel et al. 1992). In addition, it is likely that deformation of tissue components occurs without much strain of the alveolar epithelium in an intact lung during normal tidal breathing since the scaffold is stress bearing while surface tension in presence of an intact surfactant system is reduced at low lung volumes.

The basal lamina and the other components of the extracellular matrix form the scaffold to which the cellular components including alveolar epithelial cells, interstitial cells and endothelial cells are fixed via cell-matrix contacts such as focal adhesions. Although cells can execute deforming forces on surrounding extracellular matrix, it has been shown that lung mechanical properties hold only minor changes during the course of decellularization of the lung scaffold (Nonaka et al. 2014). In this context, it has been estimated that the cellular components of the inter-alveolar septa contribute little to overall lung mechanical properties such as elastic recoil and stiffness (Oeckler and Hubmayr 2008). Moreover, strain and stress acting on the extracellular matrix can be transmitted to the stress-bearing elements of the cells via cell-matrix and cell–cell contacts, such as the plasma membrane and the cytoskeleton, a mechanism which seems to be most relevant at larger lung volumes. These forces result in cellular deformation and have been estimated to amount to 5000 Pa at a focal adhesion. In this regard, Tschumperlin and Margulies reported an increase in the surface area of the epithelial basal lamina by 35% comparing lung volumes which corresponded to 42% and 100% of TLC (Tschumperlin and Margulies 1999). Therefore, at larger lung volumes the cytoskeleton and the plasma membrane of alveolar epithelial and endothelial cells might also become stress bearing due to their linkage with the basal lamina (Cong et al. 2017). Hence, these cell layers become susceptible for stress failure, e.g. upon injurious ventilation with increased tidal volumes which has been shown to result in ultrastructural signs of injury of endothelial and epithelial cells such as disruption of the cellular plasma membrane, blebbing and denudation of the basal lamina (Costello et al. 1992; Fu et al. 1992; Dreyfuss and Saumon 1998). Of note, according to these studies the type II alveolar epithelial cells appeared to be less damageable upon increased strain (Dreyfuss and Saumon 1998). Nevertheless, different mechanisms have been observed allowing cells to resist increased strain without failure. Increased strain at the cellular level might also occur under physiological conditions, e.g. upon deep inspirations, during physical exercise or sighs. Pleats of the plasma membrane unfold to accommodate to lateral tension forces. But even if breaks emerge in the plasma membrane the cells can repair these defects without undergoing cell death. Plasma membrane breaks with a diameter of less than 1 µm can be repaired by thermodynamic lateral flow of plasma membrane forming lipid bilayers in a Ca^2+^ independent way (Vlahakis et al. 2002; Cong et al. 2017). Moreover, stretching of cells has been demonstrated to activate the so-called deformation-induced lipid trafficking which includes the transfer of endogenous lipid vesicles to the corresponding break but also endocytosis of a disrupted plasma membrane and the formation of a membrane patch (Cong et al. 2017).

### Surface tension and alveolar micromechanics

The pulmonary surfactant system also contributes to pulmonary mechanics and stabilizes alveoli, in particular at lower lung volumes (Bachofen and Schürch 2001). High surface tension at the air–liquid interface has important effects on the alveolar micro-architecture and leads to a reduction in alveolar surface area by causing collapsibility of airspaces. This is counteracted by the intra-alveolar surfactant through reduction of surface tension at end-expiration. The surface active surfactant layer at the air–liquid interface not only prevents end-expiratory alveolar collapse and edema formation (Possmayer et al. 2001) but also interfacial stress. Interfacial stress in the context of high surface tension is based on fluids which oscillate on the surface of the epithelium during breathing and deform e.g. type II alveolar epithelial cells via shear forces. Hence, interfacial stress alone can result in major dysfunction of type II alveolar epithelial cells as illustrated in in vitro test systems (Hobi et al. 2012; Ravasio et al. 2011).

In a healthy lung macro- and micromechanical properties are dominated by surface tension at the alveolar air–liquid interface at low lung volumes while at larger lung volumes extracellular matrix components become stress bearing and define lung structure and mechanics (Bachofen et al. 1987; Wilson and Bachofen 1982; Bachofen and Schürch 2001). The axial system of fibers surrounding the alveolar entrance rings forms a circular lattice which surrounds the alveolar duct and balances the surface tension at the alveolar air–liquid interface (Fig. 4). The stresses resulting from surface tension in the alveoli act in a way that the inter-alveolar septa would pile up in the corner of the alveolus so that alveolar surface area decreases. Thereby the elastic and collagen fibers which surround the alveolar ducts and concentrate at the edges of septal walls are stretched. Hence, these fibers balance the forces generated by surface tension at the alveolar air–liquid interface. The intra-alveolar surfactant system, in concert with the fiber network stabilizes the alveolar surface area available for gas exchange, which would otherwise decrease with increasing surface tension (Wilson and Bachofen 1982). Filling the lung with saline abrogates the air–liquid interface and, therefore, the surface tension. This has several effects on the microarchitecture such as a relatively narrow diameter of the alveolar ducts, irregular alveolar texture with bulging capillaries and missing pleats of septa in the alveolar corners (Gil et al. 1979). In the air-filled lung with preserved surface tension the alveolar walls are flat and the capillaries do not bulge into the airspace while alveolar ducts appear to be wider. Hence, compared to saline-filled lungs, air-filled lungs have reduced alveolar surface areas as measured by design-based stereology due to the mouldering effect of surface tension (Gil et al. 1979). Increased surface tension resulting e.g. from injury of surfactant-producing type II alveolar epithelial cells is linked with a further increase of alveolar duct diameter and loss of alveolar surface area, structural alterations which might easily be misinterpreted as pulmonary emphysema (Mouded et al. 2009). These observations indicate that the alveolar surface area is a direct function of surface tension: the higher the surface tension the lower the alveolar surface area at low to intermediate lung volumes up to 80% of TLC (Bachofen et al. 1979; Bachofen and Schürch 2001). Surface tension at the air–liquid interface is reduced by surfactant to nearly zero mN/m at the end of expiration so that alveoli are stabilized and the axial system of elastic fibers is only slightly stretched at lower lung volumes (Wilson and Bachofen 1982; Bachofen et al. 1987). This interplay of surface forces and the fiber system is of high importance since it implicates that during normal breathing, e.g. ranging from 40 to 80% of TLC, the cellular components of the inter-alveolar septa like the alveolar epithelium are protected from potentially harmful mechanical stresses. At larger lung volumes, e.g. above 80% of the TLC, surface tension increases but elastic as well as collagen fibers and the epithelial basal lamina are stretched so that they now stabilize and shape the airspaces and can potentially transmit stress and strain to cells. Since these tissue components become stress bearing and their properties define mechanical characteristics at larger lung volumes the surface tension can be neglected (Wilson and Bachofen 1982; Maina and West 2006). In this context, aging has been shown to result in airspace enlargement and reduction in elastic recoil of the lung. These observations have recently been suggested to be explainable by a pure age-related redistribution of elastic and collagen fibers within the inter-alveolar septa “away from the alveolar duct” emphasizing the relevance of the spatial distribution and orientation of the fiber networks (Subramaniam et al. 2017).

### Mechanisms of alveolar micromechanics

As outlined above, the structural design of the lung suggests that inter-alveolar septa are protected from overwhelming mechanical stress and strain, at least during tidal ventilation under healthy conditions (Bachofen and Schürch 2001). This aspect is of high importance since in vitro cell culture studies have illustrated that alveolar epithelial cells are susceptible to strain induced cell damage (Tschumperlin et al. 2000; Dolinay et al. 2017), an issue which is undoubtedly of relevance in vivo in the context of ventilation-induced lung injury (VILI) (Cong et al. 2017). However, our current knowledge regarding alveolar micromechanics and the mechanics of deformation of alveoli including their walls during ventilation is still very limited (Roan and Waters 2011). This is due to limitations in spatial and temporal resolution of available imaging techniques which do not allow direct visualization of acinar micromechanics, defined as architectural and functional alterations during the respiratory cycle. Indeed, a meaningful investigation taking cellular strain in the inter-alveolar septa also into account would require electron microscopic resolution. Based on quantitative assessments of the alveolar surface area or alveolar size at different stages of the pressure–volume curve it has been estimated that the deformation and thus the strain of the alveolus in linear dimensions during tidal breathing (usually defined between 40 to 80% of TLC), is in the range of 4% (Tschumperlin and Margulies 1999; Mercer et al. 1987) to 10% (Gil et al. 1979) and can in principle even increase to 20% and more (Gil et al. 1979; Mercer et al. 1987) in case the inspiratory reserve is exhausted, e.g. during deep sighs or physical exercise (Fredberg and Kamm 2006). Fredberg and Kamm emphasized the relevance of these estimations of alveolar strain. They calculated that during lifetime the alveolar structures must cope with breathing related alveolar strain for up to 10^9^ strain cycles and further concluded: “By the standards of common engineering materials, these strains are extreme and would appear to call for tissue structures that are rather substantial” (Fredberg and Kamm 2006).

From this reasoning, the question arises how inter-alveolar septa cope with volume change related strain. In other words, what are the mechanisms by which alveoli, and above all the inter-alveolar septa adapt to respiratory cycle-related alveolar and septal strain without subjecting the alveolar epithelial cells to undue mechanical stress. Elegant studies over 30 years ago, using quasi-static conditions and vascular perfusion fixation of lung tissue at defined inspiratory and expiratory pressures during pressure–volume (PV) loops revealed that different mechanisms of septal wall deformation are involved and can result in individual alveolar volume changes during inspiration and expiration. Gil and co-workers discussed 4 mechanisms: (1) Recruitment/derecruitment of alveolar units; (2) Isotropic stretching and destretching with balloon-like changes of alveolar size; (3) Changes in alveolar shape (e.g. from dodecahedral to spherical and vice versa) which due to different geometry is linked with changes in alveolar size; and (4) Folding and unfolding of alveolar walls and accordion-like deformation which might resemble the folding and unfolding of a paper bag (Gil et al. 1979). These potential mechanisms were derived from ex vivo approaches of isolated and perfused lungs and evaluated the behaviour of the lung in a range of pressures at the airway opening from nearly zero (0.1 cm H_2_O) to up to 30 cm H_2_O (= 100% TLC) (Bachofen et al. 1987). These observations are, therefore, difficult to compare to the in vivo situation in which lung volume usually does not drop below residual volume. In the first respiratory cycles of a degassed lung, recruitment of complete alveoli during inspiration plays an important role and explains the increased hysteresis of the initial quasi-static PV loops (Bachofen et al. 1987; Bates and Irvin 2002; Carney et al. 1999). Model-based approaches using magnetic resonance imaging have provided evidence that alveolar recruitment during inspiration might be involved under physiological conditions in healthy humans (Hajari et al. 2012). However, these model predictions are in conflict with a number of other studies using direct visualization of alveoli or quantitative assessments of lung structure by means of design-based stereology to investigate alveolar micromechanics in healthy lungs. These studies did not find any evidence for intra-tidal recruitment and derecruitment of complete alveoli in a healthy lung under in vivo conditions above functional residual volume (Oldmixon and Hoppin 1991; Schiller et al. 2001; Pavone et al. 2007; Perlman et al. 2011; Sera et al. 2013; Knudsen et al. 2018; Lovric et al. 2017). In an interconnected network of alveolar walls the alveoli have been predicted to be very stable with balanced stresses acting on inter-alveolar septa as long as surface tension is reduced and harmonized between alveoli of different sizes (Fung 1975; Mead et al. 1970).

**Fig. 5:**
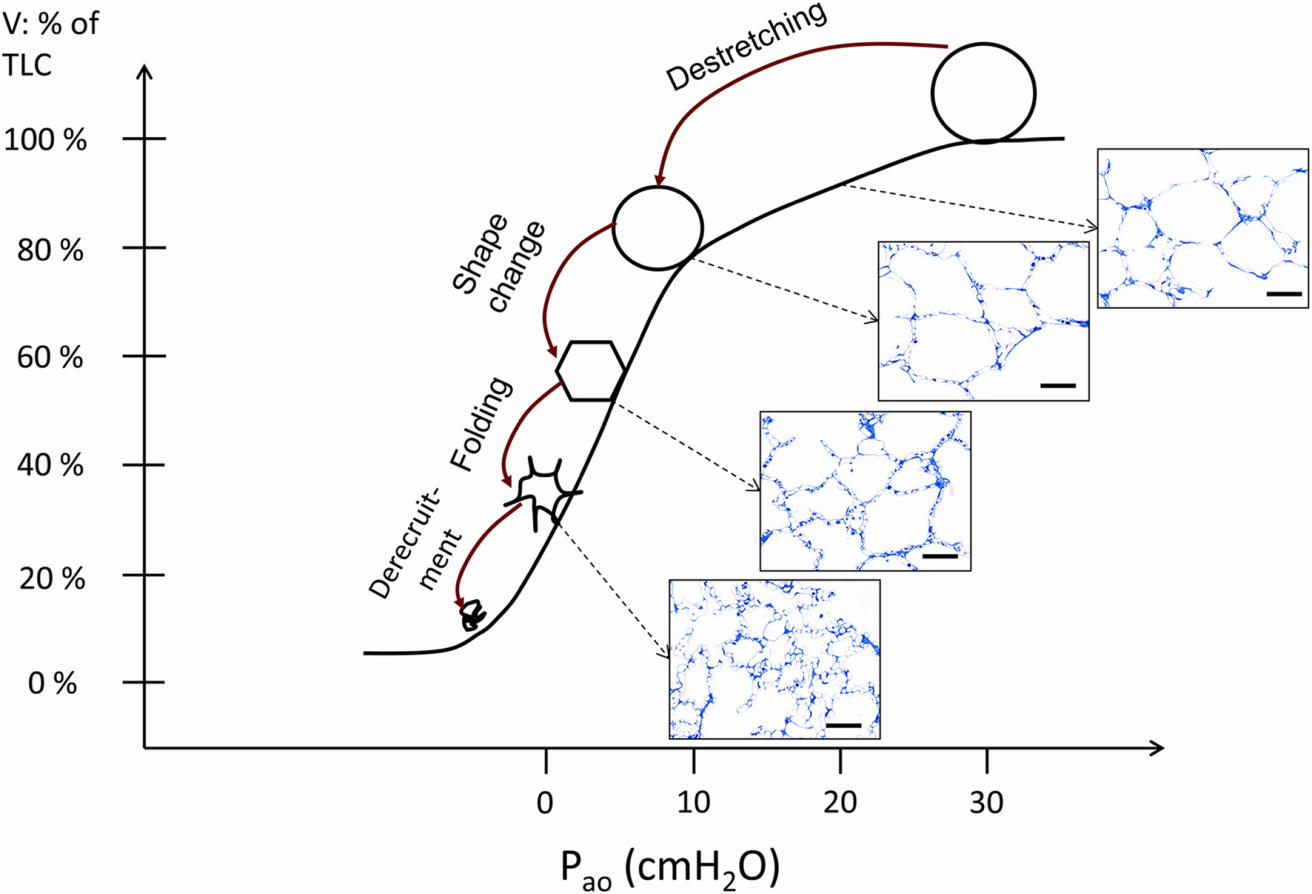
Mechanisms of alveolar micromechanics during the deflation limb of a pressure–volume curve. Four mechanisms have been suggested (Gil et al. 1979): (1) Alveolar derecruitment, (2) Isotropic (balloon-like) destretching, (3) Shape changes and (4) Folding of alveolar walls. In vivo, the lung volume usually does not drop below the functional residual volume which is above the inferior infliction point. The occurrence of alveolar derecruitment is unlikely in this range of pressures but can be observed at very low lung volumes, e.g. with negative airway opening pressures. The other 3 mechanisms are likely to occur throughout the partial PV relationship above FRC although there is good evidence that folding dominates at lower volumes while destretching is most prominent at larger volumes. Shape changes have been described to be very dominant at intermediate volumes. This depiction is based on the observations of Gil et al. (1979), Bachofen et al. (1987), Tschumperlin and Margulies (1999) and Knudsen et al. (2018). The light microscopic images were taken from histological sections of a previous study (Knudsen et al. 2018). The description provided in this image is based on evaluations of lungs fixed at different pressures during the PV loop. Isolated phenomena occurring in the septal walls such as folding, shape change or stretching have never been observed in exactly the same alveolus directly. Scale bar 50 µm

Figure 5 summarizes probable concepts of alveolar micromechanics during deflation in healthy lungs based on morphometric studies using fixed lung tissue at different stages of quasi-static PV loops (Gil et al. 1979; Bachofen et al. 1987; Oldmixon and Hoppin 1991; Tschumperlin and Margulies 1999; Knudsen et al. 2018). However, it has to be emphasised that the volume history of the lung as well as the conditions under which the lungs were studied, e.g. in vivo vs. ex vivo, are of high relevance so that conflicting results have been published. At rather low lung volumes (= decreasing airway opening pressures) the formation of pleats of inter-alveolar septal walls has been observed to occur quite frequently so that unfolding and folding of alveolar septal walls (but not complete alveolar units) appears to be of relevance for alveolar micromechanics, above all at low lung volumes. This has been shown by structural evaluations performed both under ex vivo (Tschumperlin and Margulies 1999) and in vivo conditions (Knudsen et al. 2018). Indeed, it has been demonstrated that tissue elastance during mechanical ventilation with PEEP of 1 cm H_2_O was significantly increased compared to mechanical ventilation with a PEEP of 5 cm H_2_O in healthy rat lungs. In other words, the lung gets stiffer if the pressure at the airway opening is reduced from 5 to 1 cm H_2_O. Based on the establishment of structure–function relationship, this increase in lung stiffness demonstrated a high correlation with the decrease in mean alveolar size which could be most likely attributed to the occurrence of pleats of inter-alveolar septa during deflation from 5 to 1 cm H_2_O as observed at the electron microscopic level (Knudsen et al. 2018; Tschumperlin and Margulies 1999). Of note, the number of open alveoli did not differ between lungs fixed in vivo (closed chest) at airway opening pressures of 1 and 5 cm H_2_O on expiration so that there was no evidence for derecruitment of alveolar units. Instead, a decline in surface area and alveolar volume could be linked with the formation of pleats/foldings of inter-alveolar septa. However, other investigators using a similar experimental setup (in vivo, closed chest) have questioned the relevance of this mechanism in a healthy lung since folding or crumpling of inter-alveolar septa were hardly seen between ranges of airway opening pressure of 3–16 cm H_2_O. However, the formation of pleats below this pressure range could not be ruled out (Oldmixon and Hoppin 1991). At intermediate to higher lung volumes reaching up to 100% of TLC (usually defined as lung volume at 30 cm H_2_O transpulmonary pressure), shape changes of alveolar airspaces and stretching/destretching of alveolar walls have been pointed out to occur by several investigators (Roan and Waters 2011). Gil and co-workers described changes in shape from a polyhedral to a more spherical configuration under ex vivo conditions (Gil et al. 1979). Tschumperlin and Margulies, in an ex vivo experimental setup, measured the surface area of the epithelial basal lamina during the deflation limb of a PV loop coming from 25 cm H_2_O and observed quite stable values in the range of approximately 80–40% of TLC while there was a considerable decrease in the range between 100 to 80% TLC. From these data, the authors suggested that stretching of alveolar epithelial cells is of high relevance above 80% of TLC while below 80% TLC, deformations without much change in the surface area of the basal lamina (and, therefore, stretch of alveolar epithelial cells) dominate micromechanics, e.g. unfolding/folding of septal walls or changes in shape (Tschumperlin and Margulies 1999). The overall strain in two dimensions of the basal lamina and, therefore, of the adhering epithelial and endothelial cells has been estimated to amount to 35% between 40 and 100% TLC while the range between 80 and 100% TLC contributed 80% of this overall strain (Tschumperlin and Margulies 1999). In this context, 25% strain in two dimensions of a monolayer of primary alveolar epithelial cells which might correspond to a permanent ventilation of the lung to lung volumes which are between 80% and 100% TLC, resulted in cellular injury such as endoplasmic reticulum stress and apoptosis (Dolinay et al. 2017). These findings illustrate that lung volume during ventilation does not need to exceed TLC to impose undue, potentially harmful strain on the alveolar epithelium.

Using ex vivo approaches, other investigators found evidence for recruitment and derecruitment in a range of airway opening pressures between 0 and 30 cm H_2_O (Gil and Weibel 1972; Bachofen et al. 1987) corresponding to the pressure–volume relationship over the complete range of TLC of an isolated lung. Hence, the volume history differed between those ex vivo studies which observed alveolar derecruitment and those which did not. During normal breathing and under in vivo conditions, the lung volume does usually not fall below the functional residual capacity and, therefore, the inferior infliction point of the PV loop (Salazar and Knowles 1964; Venegas et al. 1998), an aspect which is much different from ex vivo experiments mentioned above so that the occurrence of alveolar derecruitment at end-expiration is more than questionable in the range of physiological breathing. Using in vivo microscopy and the un-physiological situation of degassed but healthy dog lungs, Carney and co-workers observed alveolar recruitment till a lung volume corresponding to approximately 80% of TLC while those alveoli which were open were characterized by more or less stable individual volumes (Carney et al. 1999). Synchrotron refraction-enhanced computed tomography for direct visualization of lung acini in situ did not provide any evidence for alveolar recruitment in mice during inflation under quasi-static conditions and supported the concept of shape changes and accordion-like unfolding during inspiration (Sera et al. 2013).

Several ex vivo and in vivo studies also demonstrated that in the range of physiological breathing (e.g. transpulmonary pressure gradients below 10 cm H_2_O) volume changes predominantly take place in alveolar ducts while a smaller proportion of volume change takes place in the alveoli (Sera et al. 2013; Mercer et al. 1987; Knudsen et al. 2010, 2018). During spontaneous breathing it has been estimated from synchrotron X-ray imaging that 34% of tidal volume results in an increase of alveolar volume while the remaining 66% end up in the ductal or conducting airspaces during inspiration (Chang et al. 2015). These findings are roughly confirmed by in vivo microscopy showing that alveolar size increase only little during tidal ventilation (Schiller et al. 2001). Hence, tidal ventilation related volume changes in the alveolar compartment seem to be comparably low so that variations in linear dimensions of alveoli have been estimated to amount to 3–4%. At larger volumes, however, e.g. transpulmonary pressure gradients > 10 cm H_2_O, morphometric data suggest that 50% of volume changes during a PV loop occurs in alveolar ducts and alveoli each (Mercer et al. 1987).

## Dysfunction: lung injury and fibrosis

The properties of the elastic and collagen fiber network as well as the surface tension at the air–liquid interface in the alveolar space determine alveolar micromechanics and ultimately the lung´s mechanical properties at the organ scale. The balance of stresses within the lung parenchyma is essential for more or less homogenous ventilation of distal airspaces during the respiratory cycle under healthy conditions. Hence, in their two-dimensional model of an interconnected network of elastic elements in the lung, Mead and co-workers emphasized the fundamental role of homogeneity in ventilation to avoid mechanical stress and strain of septal walls during breathing (Mead et al. 1970). Lung pathologies resulting from acute and chronic lung injury, however, severely interfere with homogenous ventilation. Heterogeneous ventilation occurs in the context of surfactant dysfunction, alveolar collapse, intra-alveolar edema formation, lung inflammation or focal fibrotic remodeling. These pathologies have been assigned the roles of stress concentrators which might impose potential harmful stresses and strains on surrounding tissue during respiration (Mead et al. 1970; Makiyama et al. 2014). The spring model proposed by Mead et al. (1970) was based on the interdependence of hexagonally shaped alveoli which share inter-alveolar septal walls. As long as ventilation is homogenous the intra-pulmonary stresses are balanced, protecting lung units (= alveoli) from end-expiratory collapse and inspiratory overdistension. Inhomogeneity in ventilation of alveoli is linked to severe abnormalities in alveolar micromechanics due to end-expiratory collapse combined with enormous stress and intra-tidal deformation (= strain) of neighbouring alveoli including inter-alveolar septa (Mead et al. 1970; Wilson and Bachofen 1982; Makiyama et al. 2014). These predictions implicate that within an injured lung alveolar micromechanics can be locally severely altered and might become an independent trigger of lung injury progression.

Surfactant dysfunction results for example from injury of type II alveolar epithelial cells and predates the development of further signs of acute lung injury (ALI) such as formation of alveolar edema, increase in inflammatory markers or even remodelling of lung tissue (Steffen et al. 2017; Lutz et al. 2015; Lopez-Rodriguez et al. 2016; Ikegami et al. 2005; Ochs et al. 1999). Surfactant dysfunction is detrimental for the lung since it counteracts the goal of homogenous ventilation and even distribution of stresses. In addition to increases in surface tension and alveolar instability occuring at low lung volumes, differences in surface tension between different alveoli can occur resulting in intra-acinar-pressure gradients which is the basis of the phenomenon of alveolar pendelluft (see below) (Tabuchi et al. 2016). Conversely, surfactant dysfunction may also be a direct consequence of abnormal alveolar micromechanics. Mechanical but also spontaneous ventilation can be associated with undue alveolar strain and large changes of the alveolar surface area which has been shown to be responsible for alterations of the intracellular and intra-alveolar surfactant system and finally acute lung injury (Milos et al. 2017; Veldhuizen et al. 2002; Mascheroni et al. 1988). Mechanical ventilation with high tidal volumes to induce abnormally high strain of the lung results in a conversion of biophysically active large aggregates of surfactant to inactive small aggregates of surfactant within the airspaces (Veldhuizen et al. 2002). In light of these observations ALI or acute respiratory distress syndrome (ARDS) has been suggested to be primarily a disease of ventilation and altered micromechanics and atelectasis was discussed to play a central part in this pathophysiology (Albert 2012).

The relevance of abnormal alveolar micromechanics as an independent trigger of lung injury during mechanical ventilation has been well described within the last decades. In this context, the dynamic stresses and strains of lung parenchyma induced by cyclic closure and reopening of alveoli during the respiratory cycle (atelectrauma) (Muscedere et al. 1994; Steinberg et al. 2004; Chu et al. 2004) as well as overdistension of patent alveoli (volutrauma), e.g. due to heterogeneous ventilation (Retamal et al. 2014) have been shown to represent important aggravating mechanisms in ARDS. In a healthy lung the combination of volutrauma with increased pulmonary strain and atelectrauma are commonly both necessary to induce acute lung injury while in pre-injured lungs one of these factors usually suffices to aggravate lung injury (Seah et al. 2011; Nieman et al. 2015). Atelectrauma, sometimes also referred to as low volume trauma results from cyclic opening and closing of distal airspaces or even alveoli and has been shown to augment acute lung injury (Muscedere et al. 1994; Steinberg et al. 2004). During the time course of progressive VILI, increasing alveolar instability with cyclic intratidal alveolar recruitment and derecruitment (R/D) as well as edema formation has been linked to degradation of lung mechanical properties (Smith et al. 2013, 2017). In the context of atelectrauma due to surfactant dysfunction and accumulation of intra-alveolar edema, derecruited alveoli are forced to be reopened during inspiration by an entering air bubble. Based on modelling approaches including computational simulations and in vitro validation, the event of an airway reopening exerts harmful deforming stresses on epithelial cells which result during the time course of airway reopening in pulling, pushing and shearing-off of affected cells (Bilek et al. 2003; Kay et al. 2004). Volutrauma on the other hand results in overdistension of inter-alveolar septa at larger alveolar volumes in situations where stresses of the scaffold are transmitted to cells via cell-matrix contacts which then undergo stress failure. These mechanisms were recently discussed in detail in the context of both acute lung injury and pulmonary fibrosis (Cong et al. 2017). The occurrence of cellular stress failure has been documented in particular at an ultrastructural level for alveolar epithelial and endothelial cells (Dreyfuss and Saumon 1998).

The effects of ALI on alveolar micromechanics, however, go beyond the mechanisms of alveolar R/D (Gatto and Fluck 2004; Schiller et al. 2001) and alveolar overdistension (Cong et al. 2017). Using in vivo real time imaging techniques such as optical coherence tomography and intravital microscopy investigators described several phenomena which are summarized as asynchronous alveolar dynamics which occur independent from alveolar R/D (Mertens et al. 2009; Tabuchi et al. 2016). Under healthy conditions alveoli and alveolar clusters expand in synchrony with the ventilator. This means that during end-expiratory and end-inspiratory plateau phases, characterized by zero flow at the airway opening, no volume changes can be observed. After induction of acute lung injury, Mertens and co-workers (2009) observed a reduced ventilation-associated expansion in a cohort of alveoli while other alveoli were enlarged. This observation was in line with the concept of re-distribution of air within acinar airspaces and heterogeneous ventilation (Mertens et al. 2009; Schirrmann et al. 2010). Using different mouse models of ALI, Tabuchi et al. (2016) succeeded in tracking alveolar micromechanical changes during the development of ALI. Asynchronies between ventilator and alveolar dynamics were observed as early as 10 min after induction of lung injury and corresponded to the phenomenon of alveolar pendelluft. Alveolar pendelluft has been defined as an asynchronous alveolar micromechanical pattern: A cohort of alveoli increased individual volumes during the end-expiratory plateau phase while during end-inspiratory plateau pressures these alveoli decreased in alveolar volumes (Tabuchi et al. 2016). The fact that alveoli changed their size during plateau phases suggested the existence of intra-parenchymatous pressure gradients which resulted in redistribution of air. With disease progression further phenomena of alveolar asynchronies emerged which included alveolar stunning (lack of volume changes during ventilation) and inverse alveolar ventilation (volume decrease during inspiration, volume increase during expiration) which were both linked to impaired oxygenation of blood in adjoining septal wall capillaries (Tabuchi et al. 2016). Using confocal microscopy to visualize alveolar dynamics in an ex vivo rat lung perfusion model Perlman et al. investigated the alveolar interdependence between edema filled alveoli and air-filled alveoli (Perlman et al. 2011). Based on these observations, edema filled alveoli exert tethering forces on air-filled alveoli since the forces acting on the inter-alveolar septa were no longer balanced, and high surface tension dominates in the edema filled alveolus. Hence, due to the occurrence of edema filled alveoli the air-filled alveoli become prone to overdistension and, therefore, volutrauma as a result of the interdependence of alveoli which share inter-alveolar septa (Perlman et al. 2011; Wu et al. 2014). These observations provided convincing experimental evidence with respect to the model-based predictions of Mead and coworkers (1970).

## Outlook

It is evident that both the connective tissue fiber system and the surfactant system are essential and interdependent components of alveolar micromechanics (Weibel and Bachofen 1997). For alveolar physiology and pathophysiology, it is not only relevant what is going on in the alveolar wall—but also what is going on on its surface. In that sense, surfactant is certainly more than “mere paint on the alveolar wall” (Nicholas 1996). Nevertheless, despite considerable efforts over the last decades, we are still far from a comprehensive understanding of alveolar micromechanics.

The development of real-time in vivo microscopy techniques have undoubtedly advanced the understanding of alveolar micromechanics during mechanical ventilation under physiological and pathophysiological conditions as outlined above. Due to technical constraints, the access to the alveoli is, however, limited so that most studies using in vivo microscopy analyzed subpleurally located alveoli which might not be representative of the whole population of alveoli within the lung. In addition, while in vivo microscopy allows the analyses of alveolar volume changes, the mechanisms of deformation occurring within the inter-alveolar septa cannot be evaluated since the resolution is not sufficient for this purpose. Hence, if the volume change within an alveolus results in a real stretching of cells in the inter-alveolar septa or is linked with an unfolding of pleats and shape changes remains unanswered by these investigations. In-depth analyses to answer these questions would require electron microscopic resolution (for review, see Ochs et al. 2016) to visualize the basal lamina (and the epithelial cells) which might be stretched so that its surface area increases or simply unfold as ultrastructural investigations from fixed lungs suggest. Until now, however, there is no direct visual evidence whether these mechanisms are really involved during spontaneous or mechanical ventilation in a living subject.

Some of these limitations might be addressed by computational simulations (Burrowes et al. 2018). Computational modelling has the potential to advance our understanding of the acinar micromechanics and alveolar interdependence including the effects down to the cellular level. Measurements of lung mechanical properties such as elastance and compliance at the organ level, so-called macromechanics, reflect mechanisms which occur at the alveolar level such as alveolar R/D or overdistension (Knudsen et al. 2018). With this regard, computational modelling of alveolar micromechanics has been employed to investigate pathologic alterations in R/D in mice with VILI during injury progression (Smith and Bates 2013) to understand the injurious mechanical forces (Hamlington et al. 2016), and to predict the open fraction of respiratory units and alveolar distension as a function of airway pressure and disease severity (Smith et al. 2013, 2015; Knudsen et al. 2018). These empirical models of alveolar R/D are based on the assumption that at a microscopic level the lung is composed of respiratory units (e.g. alveoli) ventilated in parallel. Each alveolus has a certain elastance and viscoelasticity and the collectivity of all alveoli defines the mechanical properties at the organ scale. During the respiratory cycle, the transpulmonary pressure increases during inspiration and decreases during expiration. Depending on the surface tension and “stickiness” of the fluid alveolar lining, alveoli can derecruit (i.e. collapse) if the pressure falls below a certain limit, the alveolar closing pressure. During inspiration, however, alveoli can again be recruited in case the transpulmonary pressure exceeds a certain alveolar opening pressure (Bates and Irvin 2002). These model-assumptions were advanced in recent years and have been demonstrated to be able to reproduce empirically measured lung mechanical properties as well as structural data in several studies (Smith et al. 2013; Knudsen et al. 2018).

Spring network models were developed for simulations of the mechanics of lung parenchyma. In its original description, the spring model was composed of a two-dimensional network of springs (i.e. inter-alveolar septa) forming hexagonal (i.e. alveolar) spaces (Mead et al. 1970). This model was further developed and applied to simulate the time course and lung mechanical impairment of pulmonary fibrosis as well as pulmonary emphysema including response to lung volume reduction surgery (Bates et al. 2007; Mishima et al. 1999; Mondoñedo and Suki 2017). In addition, spring models were used to understand aspects of alveolar and alveolar-airway interdependence (Ma and Bates 2012, 2014; Ma et al. 2013a, b, 2015; Mead et al. 1970; Makiyama et al. 2014; Bates et al. 2007). It has long been understood that alveolar interdependence plays an important role in the determining strain at the level of individual septa (Mead et al. 1970; Perlman et al. 2011). However, the influence of surfactant dysfunction and derecruitment on the septal strain distribution is not well described, in particular during disease progression. In this regard, it has been proposed that surfactant dysfunction, R/D dynamics, and alveolar interdependence with increased strain play critical roles in the pathogenesis of fibrotic lung disease (Todd et al. 2015; Lopez-Rodriguez et al. 2017; Knudsen et al. 2017; Cong et al. 2017). Recent imaging studies in lungs suffering from idiopathic pulmonary fibrosis (IPF) provided evidence for instability of distal airspaces in regions of the lung which were not yet subject to fibrotic remodeling (Mai et al. 2017; Petroulia et al. 2018). Mai and co-workers used micro-computed tomography of IPF lung explants and found microatelectases in areas close to fibrotic tissue but not yet affected by fibrosis. Based on these observations microatelectases might be attributed the roles of stress concentrators which trigger disease progression including lung injury, alveolar collapse, fibrotic remodeling and collapse induration (Burkhardt 1989; Knudsen et al. 2017).
